# Spectral Tuning in Mammalian Melanopsins

**DOI:** 10.1093/molbev/msaf158

**Published:** 2025-09-25

**Authors:** Richard J McDowell, Mazie J Gatt, Saba Notash, Robert J Lucas

**Affiliations:** Centre for Biological Timing, Division of Neuroscience, School of Biological Sciences, Faculty of Biology Medicine and Health, University of Manchester, Manchester M13 9PT, UK; Centre for Biological Timing, Division of Neuroscience, School of Biological Sciences, Faculty of Biology Medicine and Health, University of Manchester, Manchester M13 9PT, UK; Centre for Biological Timing, Division of Neuroscience, School of Biological Sciences, Faculty of Biology Medicine and Health, University of Manchester, Manchester M13 9PT, UK; Centre for Biological Timing, Division of Neuroscience, School of Biological Sciences, Faculty of Biology Medicine and Health, University of Manchester, Manchester M13 9PT, UK

**Keywords:** spectral tuning, opsin photochemistry, opsin evolution, melanopsin, visual pigments

## Abstract

Melanopsin is a light-sensitive G-protein coupled receptor responsible for essential visual and non-visual light-mediated behaviors in mammals. Human melanopsin shows maximal sensitivity (λmax) in the blue region of the spectrum (∼480 nm), and available evidence suggests that this spectral sensitivity may be retained across mammals. However, melanopsin spectral sensitivity has been defined for only a small number of species, and the molecular mechanisms determining this property remain poorly understood. Here, we use heterologous action spectroscopy to determine the spectral sensitivity of melanopsins from 8 mammalian species, selected to cover diversity in retinal physiology, lighting niche, and evolutionary distance, and of engineered mutants of mouse melanopsin designed to explore mechanisms of spectral tuning. We find that melanopsin λmax varies by only 23 nm across tested mammalian species and that, within this range, it is not strongly predicted by phylogeny, retinal physiology, or lighting niche. Mutation of residues predicted to shift the electrostatic environment of the chromophore was successfully applied to produce long and short wavelength shifts in the spectral sensitivity of mouse melanopsins. However, neither natural diversity in melanopsin λmax nor the magnitude of shifts produced by mutagenesis could be adequately predicted by mechanisms of spectral tuning established in vertebrate visual or invertebrate opsins. Our data indicate that melanopsin spectral sensitivity is constrained across mammalian species via molecular mechanisms that are substantially distinct from those defined in other branches of the opsin family.

## Introduction

The amino acid composition of animal opsins determines their sensitivity to different wavelengths of light. The mechanisms by which such spectral tuning occurs in vertebrate rod and, especially, cone opsins have been extensively investigated (Hagen et al. 2023). By contrast, little is known about spectral tuning in the third major class of mammalian retinal photopigment—melanopsin. In mammals, melanopsin is expressed in intrinsically photosensitive retinal ganglion cells (ipRGCs) and plays a key role in a range of non-visual and visual functions. Melanopsin is retained across mammalian genomes, but assessment of its spectral sensitivity is currently restricted to just a few species (Lucas et al. 2001; Berson et al. 2002; Panda et al. 2005; Qiu et al. 2005; Bailes and Lucas 2013; Tsukamoto et al. 2015; Shirzad-Wasei and DeGrip 2016; Liu et al. 2023; McDowell et al. 2024). Across these species, melanopsin sensitivity peaks around 480 nm, but it remains unclear whether this is generally true for mammalian melanopsins; similarly, the critical residues for defining melanopsin spectral sensitivity remain unknown.

Recently, we used heterologous action spectroscopy to determine melanopsin spectral sensitivity for several domesticated mammalian species (McDowell et al. 2024). Here, we adapt this methodology to further explore diversity in melanopsin spectral sensitivity by applying it to a range of mammalian species covering diversity in retinal physiology, lighting niche, and evolutionary distance. Those studies confirm that melanopsin retains peak sensitivity between 474 and 497 nm across mammalian species. We then further apply the heterologous action spectroscopy method combined with site-directed mutagenesis to explore mechanisms of spectral tuning. Our findings confirm that residues responsible for spectral tuning in vertebrate and invertebrate visual opsins are relevant for defining this property in melanopsin, but that other mechanisms are also engaged.

## Results

**Fig. 1.**
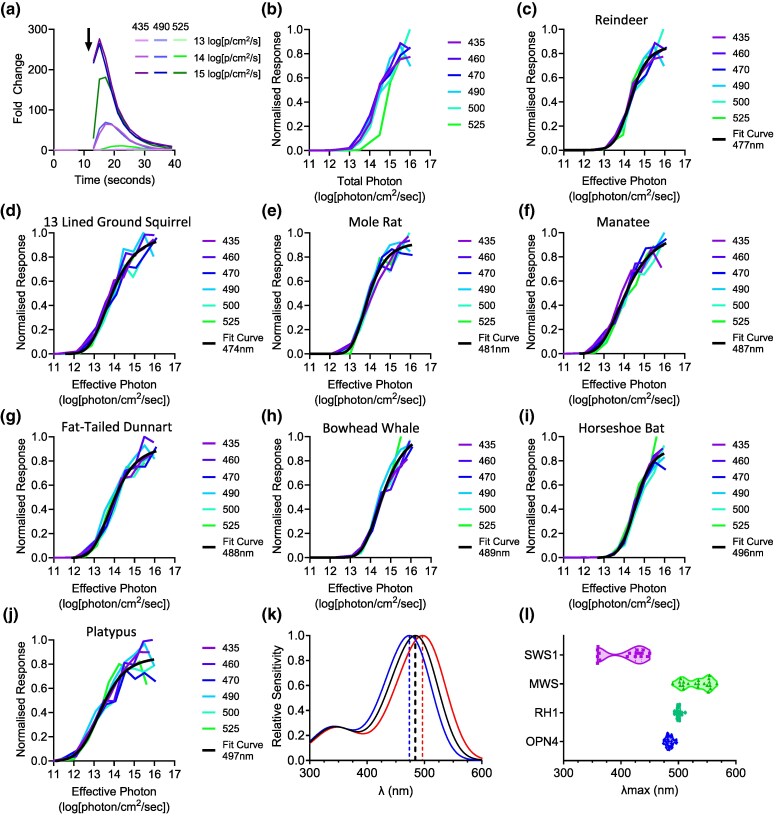
Spectral sensitivity of mammalian melanopsins. a) Example time courses for light-dependent increases in Ca^2+^ bioluminescence in HEK293 cells expressing reindeer melanopsin and aequorin in response to different intensities and spectral compositions of light (legend). The arrow indicates light pulse. b) Example irradiance response curves (IRCs) for peak luminescence for reindeer Opn4-driven responses plotted as a function of total photon flux. c–j) Example IRCs plotted as a function of effective photon flux for a putative opsin photopigment with λmax that best predicts response amplitude across wavelength and intensity for reindeer λmax = 477 nm (c), 13-lined ground squirrel λmax = 474 nm (d), mole rat λmax = 481 nm (e), manatee λmax = 487 nm (f), fat-tailed dunnart λmax = 488 nm (g), bowhead whale λmax = 488 nm (h), horseshoe bat λmax = 496 nm (i), platypus λmax = 497 nm (j). k) Govardovskii opsin photopigment templates showing the full spectral sensitivity functions for opsin pigments at the mean (black), minimum (blue), and maximum (red) λmax estimated for melanopsins in this study. Dashed lines denote λmax. l) Violin plots showing the range of λmax across each opsin photopigment class for the species investigated in this study.

We synthesized melanopsin cDNA sequences from 8 mammalian species encompassing differences in retinal anatomy, visual ecology, and/or phylogenetic distance (Table 1) and expressed them in HEK293T cells supplemented with 11-cis retinal. Cells were stimulated with 6 spectrally distinct light pulses over a range of intensities, and melanopsin-driven light responses quantified using the luminescent Ca^2+^ reporter Aequorin. As expected, response amplitude varied as a function of stimulus intensity and spectral composition (shown for a representative example, Fig. 1 a, b). Melanopsin spectral sensitivity was then estimated using a bootstrap modeling approach that determines which value for opsin peak spectral sensitivity (λmax) best predicts response amplitude across wavelengths and intensities (McDowell et al. 2024). Expressing intensity as a function of effective photon flux for this nominal opsin normalized intensity response curves across wavelengths (Fig. 1c). Across all 8 species, predicted melanopsin λmax calculated according to this method fell within the range 474 to 497 nm (Fig. 1c-j).

**Table 1:** Species chosen for melanopsin characterization and notable features

Common name	Binomial name	Notable feature
Horseshoe bat	*Rhinolophus ferrumequinum*	ipRGCS comprise 16% of Retinal ganglion cells (Jeong et al. 2018)
Mole rat	*Nannospalax ehrenbergi*	ipRGCS comprise 90% of Retinal ganglion cells (Hannibal 2021)
13-Lined ground squirrel	*Ictidomys tridecemlineatus*	Strongly diurnal, highly cone-rich retinas, low lens permeability below 410 nm (Kryger et al. 1998; Chou and Cullen 2011; Klapoetke et al. 2014)
Bowhead whale	*Balaena mysticetus*	Aquatic mammal, unusual lighting niche, rod monochromat (Shanmugam and Ahn 2007; Meredith et al. 2013)
West Indian manatee	*Trichechus manatus*	Aquatic mammal, unusual lighting niche (Shanmugam and Ahn 2007)
Reindeer	*Rangifer tarandus*	Unusual lighting niche, spectrally tunes tapetum lucidum (Fosbury and Jeffery 2022)
Platypus	*Ornithorhynchus anatinus*	Monotreme, evolutionarily distinct (Zhou et al. 2021)
Fat-tailed dunnart	*Sminthopsis crassicaudata*	Marsuipial, evolutionarily distinct (Upham et al. 2019)

Including published data for humans and domestic mammals (Table 2) produced a group mean λmax for all known mammalian melanopsins in the presence of 11-cis retinal of 484.4 nm (Fig. 1k). The range of λmax across species at 23 nm, from 474 ± 1.2 nm (13-lined ground squirrel, *Ictidomys tridecemlineatus*) to 497 ± 1.6 nm (platypus, *Ornithorhynchus anatinus*), is similar to that for rod opsins in this group (RH1 range = 19 nm from published work, see supplementary table S1, Supplementary Material online, Fig. 1l), and much smaller than for their cone opsin classes (SWS1 range = 359 to 450 nm for mouse/rat and cat respectively; medium wave sensitive (MWS) range =490 to 567 nm for Mongolian gerbil and crab-eating macaque, respectively).

**Table 2:** Spectral sensitivities of mammalian melanopsins

Common name	Binomial name	Opn4 λmax (nm)
13-Lined ground squirrel	*Ictidomys tridecemlineatus*	474 ± 1.2
Striped mouse	*Rhabdomys pumilio*	476 ± 0.9
Reindeer	*Rangifer tarandus*	477 ± 1.0
Golden hamster	*Mesocricetus auratus*	479 ± 0.7
Mouse	*Mus musculus*	480 ± 1.1
Human	*Homo sapiens*	481 ± 1.1
Mole rat	*Nannospalax ehrenbergi*	481 ± 1.2
Rat	*Rattus norvegicus*	481 ± 1.1
Horse	*Equus caballus*	482 ± 0.9
Degu	*Octodon degus*	482 ± 1.2
Crab-eating macaque	*Macaca fascicularis*	483 ± 1.2
Cow	*Bos taurus*	484 ± 1.1
Sheep	*Ovis aries*	484 ± 1.1
Manatee	*Trichechus manatus*	487 ± 1.0
Cat	*Felis catus*	488 ± 1.0
Dog	*Canis lupus familiaris*	488 ± 0.9
Fat-tailed dunnart	*Sminthopsis crassicaudata*	488 ± 1.1
Rabbit	*Oryctolagus cuniculus*	488 ± 0.7
Bowhead whale	*Balaena mysticetus*	489 ± 1.2
Gerbil	*Meriones unguiculatus*	491 ± 1.0
Horseshoe bat	*Rhinolophus ferrumequinum*	496 ± 1.4
Platypus	*Ornithorhynchus anatinus*	497 ± 1.7
**Average**	**…**	**484.4**

Previously published values (McDowell et al. 2024) are underlined.

**Fig. 2.**
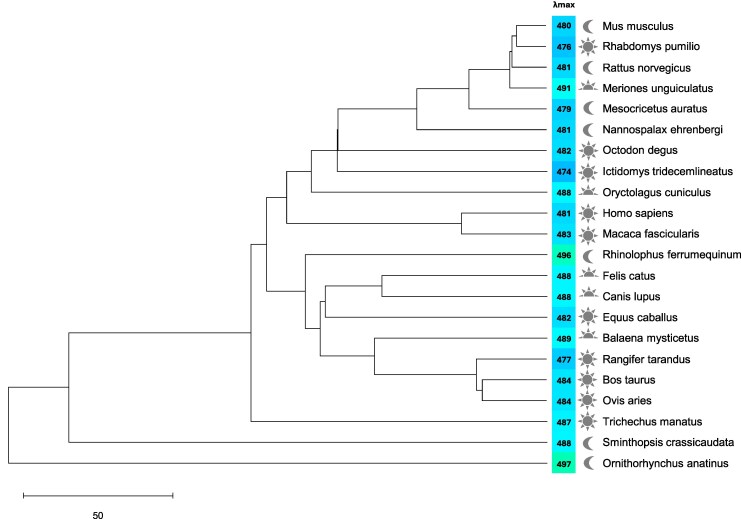
Phylogenetic relationship between species used in this study with corresponding melanopsin λmax. Topology is based upon species relationships as determined by Upham and colleagues (Upham et al. 2019). Species divergence times were determined by TimeTree5 (Kumar et al. 2022). Scale bar shows 50 mya. λmax text box is color coded to approximate the appearance of that wavelength to a human observer. Sun, half-sun, and moon symbols indicate whether the animal is diurnal, crepuscular, or nocturnal, respectively.

Differences in melanopsin λmax across mammalian species, to the extent they existed, were not strongly determined by phylogenetic relationship or visual ecology (Fig. 2). In general, more closely related species did have similar λmax, but a single order (Rodentia) encompassed nearly the full range of available λmax (476 nm to 491 nm). A moderate red-shift was observed for the evolutionarily distant platypus (*Ornithorhynchus anatinus,* λmax =497 nm), but the marsupial fat-tailed dunnart λmax (*Sminthopsis crassicaudata,* λmax =488 nm) was close to the average. Thirteen-lined ground squirrel (*Ictidomys tridecemlineatus*) and gerbil (*Meriones unguiculatus*), both of which are day active and live in prairie/steppe grassland environments, had divergent melanopsin λmax (474 and 491 nm, respectively). Moreover, the aquatic mammals tested, manatee (*Trichechus manatus,* λmax = 487 nm) and bowhead whale (*Balaena mysticetus,* λmax = 489 nm) had λmax close to the average for all mammalian species despite the spectral filtering properties of water (Jerlov 1977; Fasick et al. 2021).

**Fig. 3.**
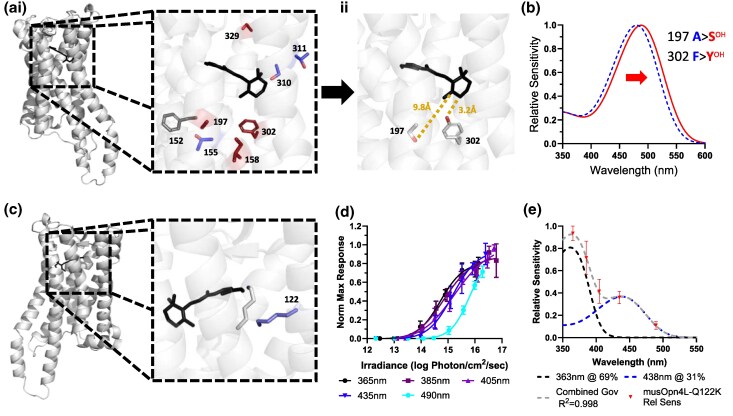
Mutation of residues near the retinal binding pocket of mouse melanopsin results in a spectral shift. a) Homology model of mouse melanopsin constructed against Squid rhodopsin crystal structure (PDB 2z73). (ai) Inset shows retinal binding pocket and relative positions of non-conserved polar amino acids. Residue color indicates the expected shift from polar residue substitution. Red = red-shift, blue = blue-shift, gray = no change expected. (aii) Retinal binding pocket for homology model of mouse melanopsin with A197S, F302Y mutations. Distance from the side chain of residues to the β-ionone ring is denoted by dashed orange lines. Homology templates and models were built in SWISS-MODEL, followed by visualization in PyMol. b) Govardovskii nomogram templates showing the relative sensitivity of WT mouse Opn4L (λmax = 480 nm, blue dashed line) and mouse Opn4L-A197S-F302Y ((λmax = 488 nm, red line). c) Homology model of mouse Opn4L-Q122 K mutant constructed against Squid rhodopsin crystal structure (PDB 2z73). Inset shows the retinal binding pocket and relative position of K122 (blue) to the Retinal Schiff Base. d) Average irradiance response curves (IRCs) for peak luminescence for mouse Opn4L-Q122 K responses plotted as a function of total photon flux. *N* = 4. e) Best fit model for relative wavelength sensitivity for mouse Opn4L-Q122 K. Relative wavelength sensitivity data (red) is best described (*R*^2^ = 0.998) by a summed nomogram (gray) comprising Govardovskii template nomograms at 363 nm (black) and 438 nm (blue) (Govardovskii et al. 2000).

As a first step to exploring the structural determinants of melanopsin spectral sensitivity, we combined the mammalian melanopsin dataset with published information for amino acid sequence and λmax in non-mammalian vertebrates. According to the established understanding of spectral tuning, residues most likely to determine wavelength sensitivity are near the retinal chromophore. We therefore generated homology models for melanopsin structures to identify residues close to the retinal binding pocket using SWISS-MODEL (Waterhouse et al. 2018) against Squid Rhodopsin (Structure ID: 2z73) (Fig. 3a). The highest confidence regions of the homology structures were in and close to the trans-membrane domains, which included the majority of residues close to the retinal binding pocket. Based upon these models, we identified all residues for which the functional group or side chain was predicted to lie within 10Å of either the β-ionone ring or the Schiff base lysine of the retinal chromophore (supplementary table S2, Supplementary Material online).

In vertebrate visual opsins, electrostatic tuning via gain or loss of polar residues around the chromophore, the “OH-rule,” is particularly important in spectral tuning (Sekharan et al. 2012). Polar residues (Serine, Tyrosine, or Threonine) near the β-ionone ring cause red-shifts, while those close to retinal Schiff base (RSB) produce blue-shifts in λmax. A number of putative chromophore-adjacent residues showed variation in the appearance of polar side chains across the melanopsin dataset (Table 3; Fig. 3a). However, attempts to relate these to differences in λmax using the predicted effects on electrostatic environment were largely unsuccessful. Neutral alanine (in place of charged serine) at position 310 is predicted to blue-shift λmax and indeed is found in short-wavelength-shifted melanopsins from striped mouse (*Rhabdomys pumilio*) and Opn4x subtypes in non-mammalian vertebrates, which are blue-shifted compared to Opn4 m subtypes in Chicken, Medaka, and Zebrafish (supplementary table S3, Supplementary Material online) (Torii et al. 2007; Davies et al. 2011; Fujiyabu et al. 2023). Conversely, melanopsin λmax diverged by as much as 19 nm across species with no deviation in amino acid composition across these residues (reindeer, *Rangifer tarandus*, λmax = 477 nm vs. horseshoe bat, *Rhinolophus ferrumequinum*, λmax = 496 nm), implying that changing these residues is not necessary to produce alterations in λmax. Nor did amino acid residue changes at these sites produce alterations in λmax in many cases. For example, mole rat (Nanno*spalax ehrenbergi*) and mouse (*mus musculus*) have very similar λmax but differ in polar residue composition across sites 158 and 329.

**Table 3:** Non-conserved OH-sites within 10Å of retinal across mammalian melanopsins used in the study

Species	λmax (nm)	Red shift	Amino acid # (mouse Opn4L)							
		(vs. mouse)	152	155	158	197	302	310	311	329
Mouse	480 ± 1.1	∼	F	T	I	A	F	S	T	S
13 LG Squirrel	474 ± 1.2	−6	F	**S"**	I	A	F	S	I*	S
Striped mouse	476 ± 0.9	−4	F	T	I	A	F	A*	T	S
Reindeer	477 ± 1.0	−3	F	T	I	A	F	S	T	**N^**
Hamster	479 ± 0.7	∼	F	T	I	A	F	S	T	S
Human	481 ± 1.1	∼	F	**S"**	I	A	F	S	A*	S
Mole rat	481 ± 1.3	∼	**S"**	T	**T^**	A	F	S	T	**N^**
Rat	481 ± 1.1	∼	F	V*	I	A	F	S	T	S
Horse	482 ± 0.9	∼	F	T	I	A	F	S	V*	**N^**
Degu	482 ± 1.2	∼	F	T	I	A	F	S	T	**N^**
Crab-eating macaque	483 ± 1.2	3	F	**S"**	I	A	F	S	A*	S
Cow	484 ± 1.1	4	F	T	I	A	F	S	T	**N^**
Sheep	484 ± 1.1	4	F	T	I	A	F	S	T	**N^**
Manatee	487 ± 1.1	6	F	T	I	A	F	S	T	**N^**
Cat	488 ± 1.0	8	F	T	I	A	F	S	I*	**N^**
Dog	488 ± 0.9	8	F	T	I	A	F	S	A*	**N^**
Fat-tailed Dunnart	488 ± 1.2	8	F	T	I	**S^**	F	S	T	**N^**
Rabbit	488 ± 0.7	8	F	**S"**	I	A	F	S	T	**N^**
Bowhead whale	489 ± 1.2	9	F	T	I	A	F	S	T	**N^**
Gerbil	491 ± 1.0	11	F	T	I	A	F	S	T	**N^**
Horseshoe bat	496 ± 1.4	16	F	T	I	A	F	S	T	**N^**
Platypus	497 ± 1.6	17	F	T	I	**S^**	**Y^**	S	V*	**N^**
**Consensus**	**…**	**…**	**F**	**T**	**I**	**A**	**F**	**S**	**T**	**N**
Distance to *N* in RSB	…	…	9.9	15.4	18.1	16.4	12.3	12.1	16.6	9.4
Distance to β-ionone	…	…	9.6	10	9.9	9.8	3.2	2.5	8.7	16.4
5 Site rule?	…	…	N	N	N	Y	Y	Y	N	N

Amino acid substitutions are compared against mouse melanopsin. ∼ indicates no change in λmax for opsin. * indicates expected blue-shift from amino acid substitution. ^ indicates expected red-shift from amino acid substitution. " indicates no expected changes in λmax from amino acid substitution.

The failure of the “OH-rule” to predict melanopsin λmax has two non-exclusive interpretations. On the one hand, our homology model of the melanopsin chromophore binding pocket may be inaccurate, leading us to interrogate the wrong residues. Alternatively, additional spectral tuning mechanisms may be engaged that complement/extend the impact of polar residues close to the chromophore. Steric hindrance of the polyene chain and β-ionone ring of the retinal chromophore by nearby amino acids has been proposed as one such additional spectral tuning mechanism (Ernst et al. 2014). One way to distinguish between these alternatives is to ask if mutagenesis of residues predicted to alter spectral sensitivity according to the OH-rule and based on our structural model successfully shifts λmax. Across the mammalian melanopsins, the platypus has the longest wavelength λmax and differs from the mouse at 2 residues predicted to cause a red-shift (A197S and F302Y). Sites 197 and 302 are part of the “5-site rule” of critical spectral tuning residues in MWS cone opsins (corresponding to sites 197, 214, 302, 310, and 333 in mouse melanopsin) and have been shown to act additively to shift λmax (Yokoyama and Bernhard Radlwimmer 1998; Yokoyama et al. 2008; Sekharan et al. 2012). To test the hypothesis that composition at these sites impacts λmax, we mutated mouse melanopsin to insert A197S and F302Y from platypus melanopsin (Fig. 3a and b). These mutations resulted in a modest, 8 nm, red-shift (Fig. 3b). We did not conduct mutagenesis to test the effect of each mutation individually, as this would likely produce a very small spectral shift at the limits of detection for our experimental setup. This result provides confidence that we are identifying at least some of the residues defining the electrostatic environment of the chromophore binding pocket, but the small shift observed indicates that indeed some other mechanism(s) contribute to the 17-nm divergence between mouse and platypus λmax. We next queried whether any remaining spectral tuning residues known from vertebrate visual opsins may explain the spectral sensitivities observed in melanopsins (supplementary tables S4 and S5, Supplementary Material online). For the majority of vertebrate tuning residues, melanopsins displayed conservation of amino acids. In most cases where melanopsin amino acid substitutions were present, the substitutions were not those found in vertebrate opsins. There were only 2 residues which matched vertebrate spectral tuning residues: L81F in fat-tailed dunnart Opn4 (BovRho# 49) and A146G in horseshoe bat Opn4 (BovRho# 114).

We next asked whether understanding of spectral tuning in invertebrate opsins may provide greater insight, on the basis that melanopsins are more closely related to invertebrate rhabdomeric (r-)opsins (Provencio et al. 1998). A number of residues have been implicated in defining wavelength sensitivity in invertebrate opsins (supplementary tables S6 and S7, Supplementary Material online). For most of these sites, melanopsins retained conserved amino acid residues across species. At the majority of sites where amino acid substitutions had occurred in melanopsin, the amino acid residues exchanged were not those implicated in shifting λmax in invertebrates. This leaves the contribution of these sites to spectral tuning unknown. Only one invertebrate opsin tuning site was also present in melanopsin (F126 V in horseshoe bat Opn4 (BovRho# 94)), while the amino acid substitutions at the remaining sites did not correlate to the spectral shifts we observe across melanopsins.

To test the hypothesis that invertebrate spectral tuning mechanisms could in principle apply to melanopsins, we chose a single tuning residue from invertebrates predicted to produce a large shift in λmax. Insect r-opsins shift from visible light to UV sensitivity when lysine is present at position 90 (Kitamoto et al. 2000; Salcedo et al. 2003; Spaethe and Briscoe 2005; Henze et al. 2012; Hu et al. 2014; Sakai et al. 2017). The insertion of positively charged lysine at this position is thought to destabilize protonation of the retinal Schiff base and thus strongly blue-shift λmax. Predicted structure homology modeling of melanopsin indicated that the insertion of lysine at this position (Q122K) would also place it very close to the RSB (Fig. 3c). Mutagenesis of mouse melanopsin confirmed that the Q122K substitution produced a large increase in UV sensitivity (Fig. 3d and e). Responses across spectrally distinct stimuli could not be adequately predicted by a single pigment at any λmax (best *R*² = 0.66) (Govardovskii et al. 2000). Given the putative role of this residue in RSB protonation, we wondered whether the spectral sensitivity of this pigment could be better explained by a mixture of pigments in protonated versus deprotonated forms. Indeed, the data were well approximated by the sum of 2 Govardovskii templates (λmax 1 = 363 nm (69%), λmax 2 = 438 nm (31%), and *R*^2^ = 0.998) (Fig. 3e; Govardovskii et al. 2000).

## Discussion

Applying heterologous action spectroscopy to melanopsins from diverse mammalian species provides support for the hypothesis that this pigment's spectral sensitivity is relatively invariant across species. Combining our new data with previously published works reveals that across all 22 characterized mammalian melanopsins, λmax lies between 474 and 497 nm. This is true also for the few non-mammalian Opn4 m melanopsins for which information is available. A note of caution to this conclusion is that these values reflect melanopsin in the presence of 11-cis retinal, and we know much less about the spectral sensitivity of its other thermally stable states with all-trans and 7-cis retinal (Matsuyama et al. 2012; Emanuel and Do 2023). With that caveat in mind, our data indicate that melanopsins show equivalent spectral sensitivity conservation to the rod opsins, which are considered notably stable across mammals (Hagen et al. 2023). That melanopsins exhibit lower spectral variance than cone opsins may be surprising in light of recent analysis revealing that melanopsins display the highest sequence diversity of all opsin classes across mammals (Upton et al. 2021). However, the majority of this sequence variation arises from mutations in the N- and C-termini of the protein, which are most associated with changes to protein stability and signaling kinetics rather than spectral tuning (Blasic et al. 2014; Tsukamoto et al. 2015; Upton et al. 2021).

The retention of λmax at ∼480 nm across melanopsins implies a strong evolutionary constraint. Comparison of melanopsin λmax with habitual light environment does not obviously support the conclusion that this reflects the need to optimally absorb light at particular wavelengths. In principle, retaining peak sensitivity to 480-nm light would ensure effective activation by the spectral composition of light at dawn and dusk (Asakawa and Ishikawa 2015; Fasick et al. 2021). However, if this were the driving force for fixing melanopsin spectral sensitivity, we would expect this parameter to respond to changes in the habitual light environment. In fact, both bowhead whale (*Balaena mysticetus*) and west Indian manatee (Trichechus manatus) retain λmax close to the mammalian average, despite living underwater where the spectral composition of light differs from terrestrial mammals (Jerlov 1977; Shanmugam and Ahn 2007; Fasick et al. 2021). Similarly, reindeer melanopsin does not exhibit blue-shift in λmax despite reindeer inhabiting high arctic environments, where the spectral composition of daylight is blue-shifted (∼450 nm) during winter twilight (Fosbury and Jeffery 2022).

An alternative potential explanation for the stability of melanopsin λmax is that it reflects the need to minimize noise associated with thermal isomerization. Modeling of the melanopsin isomerization mechanism suggests that thermal isomerization would increase for pigment with λmax <470 nm (Rinaldi et al. 2014), while spectral shifts to longer wavelengths increase such dark noise in other opsins (Ala-Laurila et al. 2004; Luo et al. 2011; Gozem et al. 2012). Thermal isomerization could meanwhile be especially problematic for melanopsin because of the high efficiency of its phototransduction cascade. ipRGCs contain rather little melanopsin (pigment density at least ∼1,000 fold lower than rod opsin in vertebrate rods), meaning that they capture few photons and employ strong signal amplification (Do et al. 2008). Thermal isomerization could be strongly selected against in this scenario because of its potential to increase dark noise.

Our comparative sequence analysis and functional assessment of targeted mutants have provided partial success in understanding the molecular mechanisms constraining melanopsin λmax. The spectral sensitivity of opsin photopigments is thought to be determined by the amount of energy required to shift the retinal chromophore from the ground to the excited state (Sekharan et al. 2012; Ernst et al. 2014; Collette et al. 2018). Chromophore activation to the excited state involves delocalization of electrons and a shift of positive charge toward the β-ionone ring of retinal. Increased delocalization of electrons in the ground state acts to decrease the dipole moment between the ground and activated state, and red-shift the spectral sensitivity of the photopigment. The extent of ground-state electron delocalization, and thus λmax, is determined by the amino acids in the vicinity of the retinal binding pocket. Electron delocalization is influenced via three mechanisms: (i) protonation of the retinal Schiff base; (ii) changes to the electrostatic environment of the chromophore via polar or charged amino acid residues (Wang et al. 2012; Orozco-Gonzalez et al. 2019); (iii) alteration of the planarity of the β-ionone ring relative to the chromophore, whereby increased planarity causes red-shift (Ernst et al. 2014).

The strong blue-shift produced by insertion of lysine at position 122 (90 BovRho#) in mouse melanopsin supports the conclusion that RSB protonation is indeed an important determinant of melanopsin spectral sensitivity. Introduction of such a positive charge at this location close to the RSB is predicted to increase the likelihood that it remains deprotonated. Opsins with deprotonated RSB show UV sensitivity, and indeed, the Q122K mutant of mouse melanopsin was most sensitive to UV light. Moreover, the resultant spectra were best described by the summed total of two Govardovskii template nomograms (Govardovskii et al. 2000). Similar dual template spectra have been observed for other opsins with lysine at this position (Salcedo et al. 2003; Devine et al. 2016; Sakai et al. 2017) and interpreted as evidence of incomplete RSB protonation producing separate pools of pigment sensitive to visible and UV light (Devine et al. 2016). The hypsochromatic shift of the putative protonated fraction of pigment in the Q122K mutant relative to wild-type melanopsin (438 nm vs. 480 nm) is similar to the blue-shift observed in other opsins with insertion of lysine at this site (Devine et al. 2016). It is likely that insertion of the positively charged lysine residue alters the charge distribution around the retinal binding pocket, leading to altered spectral tuning for the protonated pigment fraction.

The wider electrostatic environment of the chromophore is also clearly an influence on melanopsin λmax. This determinant of opsin spectral sensitivity is most clearly captured in the “OH-rule”, by which the electrostatic effects of polar hydroxyl (OH)-bearing amino acids (Serine, Tyrosine, or Threonine) in the retinal binding pocket predict spectral sensitivity of vertebrate visual opsins (Sekharan et al. 2012). We find examples from our comparative analysis of putative “OH-rule” residues predicting wavelength sensitivity (replacement of serine at 310 with alanine associated with bluer λmax). Moreover, we were able to employ this approach to propose residues at positions 197 and 302 as origins of the long-wavelength bias of platypus with respect to mouse melanopsin and show by mutagenesis that this is partially true. However, there were many failures of “OH-rule” predictions. The red shift of A197S F302Y mouse melanopsin was small compared to that of the native platypus melanopsin. Generally, the presence of polar residues at these loci was a poor predictor of spectral tuning, as e.g. horseshoe bat melanopsin exhibited similar spectral sensitivity to platypus melanopsin yet lacked polar residues at positions 197 and 302. Further, chicken Opn4 m has the same amino acid residues as platypus melanopsin at all 8 putative “OH-rule” positions identified in this study, but a λmax of only 484 nm (Table 3, supplementary table S3, Supplementary Material online). Indeed, across mammalian melanopsins λmax varied by as much as 19 nm for pigments with identical amino acid residues across all “OH-rule” positions (reindeer vs. horseshoe bat).

The failure of the “OH-rule” to adequately predict melanopsin λmax in this work from either comparative analysis or mutagenesis studies highlights the need for further structural analysis. On the one hand, such structural information could reveal that the homology model approach used here to predict spectral tuning residues abutting the chromophore is inadequate. On the other hand, taken on face value, our analysis indicates that the final mechanism of spectral tuning, steric hindrance of the chromophore by the combination of non-polar residues in the chromophore binding pocket, makes a substantial contribution to defining melanopsin λmax (Ernst et al. 2014). In this case, high-resolution structural analysis of melanopsin would allow for more accurate prediction of the tuning residues important for conferring these steric constraints.

In the absence of a solved structure for melanopsin, the approach used in this study of *in silico* homology modeling combined with mutagenesis and heterologous action spectroscopy is the best way to determine spectral tuning rules governing melanopsin. Several research groups have successfully used this method to identify spectral tuning residues for melanopsin. Tennigkeit et al. (2019) and Wijayaratna et al. (2024) each used homology modeling with hybrid quantum mechanics and molecular mechanics (QM/MM) simulations to identify several spectral tuning sites in mouse melanopsin that induced a blue-shift in λmax. However, these sites showed amino acid conservation across the melanopsins screened in our study. Further research into the mechanisms underpinning melanopsin spectral tuning is needed in order to gain a more accurate prediction of melanopsin spectral sensitivity.

## Materials and Methods

### Recombinant Cloning of Animal Opsins

The following melanopsin open reading frame coding sequences were accessed from NCBI Genbank (Sayers et al. 2024) and Ensembl (Ensembl release 113) (Dyer et al. 2025) databases: 13-lined ground squirrel (*Ictidomys tridecemlineatus*) Opn4, XM_021720398; Fat-tailed dunnart (*Sminthopsis crassicaudata*) Opn4, ABD38715.1; Horseshoe bat (*Rhinolophus ferrumequinum*) Opn4, ENSRFET00010033609.1; Mole rat (Nanno*spalax ehrenbergi*) Opn4, AM748539; West Indian manatee (*Trichechus manatus*) Opn4, XM_023740472; Reindeer (*Rangifer tarandus*) Opn4, JAHWTM010000014.1; Platypus (*Ornithorhynchus anatinus*) Opn4, XM_039911324.1. Bowhead whale (*Balaena mysticetus*) Opn4, bmy_16888, was accessed from the Bowhead Whale Genome Resource (Keane et al. 2015). Mutant mouse (*Mus musculus*) Opn4L sequences were modified in silico from NM_013887.2 using SnapGene software (www.snapgene.com). All melanopsin sequences were tagged with the 1D4 epitope (TETSQVAPA) at the C-terminus. Gene sequences were synthesized using ThermoFisher GeneArt Gene Fragment synthesis and TwistBio Gene Fragment synthesis, with codon optimization where necessary for synthesis. Gene sequences were inserted into the multiple cloning site of the pcDNA3 plasmid vector (Invitrogen, Cat# V79020) using NEBuilder HiFi Assembly (New England Biolabs, Cat# E2621S).

### Heterologous Action Spectroscopy

HEK293T cells (CRL-11268, American Type Culture Collection) were cultured in Dulbecco's modified Eagle's medium (4.5 g l^−1^ D-glucose, sodium pyruvate, and L-glutamine with 10% fetal calf serum; DMEM) (Merck, Cat# D6429). Cells were transiently transfected with 500 ng opsin plasmid expression vectors and 500 ng luminescent Ca^2+^ indicator mtAequorin (as in Bailes and Lucas 2013) using lipofectamine 2000 (Invitrogen, Cat# 11668027) and incubated overnight with 10 µM 11-cis-retinal (National Eye Institute, National Institutes of Health). Prior to stimulation, cells were incubated with 10 µM Coelenterazine-h (Promega, Cat# S2011) in the dark for 2 h. Luminescence was recorded in a plate reader (Optima FLUOStar, BMG) modified with a fiber optic to allow “in-well” light stimulation via an external light source (CoolLED pe-4000, CoolLED). Luminescence recordings were sampled at a temporal resolution of 2 s per time point. Baseline luminescence was recorded for 10 s. Unless otherwise indicated, cells were stimulated with light pulses (1 s) of varying intensities (11 to 16 log photon/cm^2^/sec total photon flux) at one of 6 different wavelengths (435 nm, 460 nm, 470 nm, 490 nm, 500 nm, and 525 nm). Cells expressing musOpn4L-Q122 K were stimulated with 1-s light pulses ranging in irradiance from 12 to 17 log photon/cm^2^/sec total photon flux at one of 5 wavelengths (365 nm, 385 nm, 405 nm, 435 nm, and 490 nm).

### Calculation of Opsin Photon Sensitivity Peaks

To calculate the peak spectral sensitivity for most opsins, we used a nonlinear optimization strategy as previously described (McDowell et al. 2024).

For musOpn4L-Q122K, an alternative fitting approach was used. For each irradiance at each wavelength, the maximum fold change response was calculated. These values were used to construct irradiance response curves (IRCs) via fitting a four-parameter sigmoidal curve for each wavelength using Prism 10 (GraphPad). The relative sensitivity of each wavelength was calculated by dividing the EC50 values for each wavelength by the lowest EC50 value. The average relative sensitivity data were then fit using a nonlinear least-square method to either a single Govardovskii template nomogram or the summed total of 2 Govardovskii template nomograms (Govardovskii et al. 2000). The Govardovskii template nomogram formula for A1-based photopigments is the summed total of the α-band and β-band equations below:

α-Band for A1-based pigments:


$$S(x)=\frac{1}{\exp\;[A(a-x)]+\exp\;[B(b-x)]+\exp\;[C(c-x)]+D}$$

where *x* = λmax/λ, *A* = 69.7, *a* = 0.8795 + 0.0459 × exp[-λmax − 300)^2^/11940], *B* = 28, *b* = 0.922, *C* = −14.9, *c* = 1.104, and *D* = 0.674

β-Band for A1-based pigments:


$$S_{\beta }(\lambda )=A_{\beta }\times \exp\;{\left\{-\left[\frac{\left(\lambda -\lambda_{m\beta }\right)}{b}\right]\right\}}^{2}$$

where *A_β_* = 0.26, *λ*_mβ_ = 189 + 0.315 × λmax [nm], *b* = -40.5 + 0.195 × λmax [nm].

### Sequence and Structure Comparison of Melanopsins

Phylogenetic tree generation used topology based upon species relationships as determined by Upham et al. (Upham et al. 2019). Species divergence times were determined by TimeTree5 (Kumar et al. 2022). Tree was visualized using Molecular Evolutionary Genetics Analysis (MEGA) X (Kumar et al. 2018).

Structural homology models of each melanopsin were generated using SWISS-MODEL (Waterhouse et al. 2018) using the crystal structure of squid rhodopsin (Structure ID: 2z73, (Murakami and Kouyama 2008)). The resulting homology models had a global model quality estimate (GMQE) of 0.44 and a qualitative model energy analysis (QMEAN) score of 0.71. The GMQE and QMEAN scores for squid rhodopsin (2z73) were higher than for any other available structure. Homology models were imported into PyMOL (Schrödinger) and aligned with Squid Rhodopsin (Structure ID: 2z73) to estimate the relative position of amino acids to the β-ionone ring and protonated Schiff base nitrogen of the retinal chromophore. Melanopsin sequences were aligned using Clustal Omega (Sievers and Higgins 2018). Residues within 10Å of the retinal molecule were selected as potential spectral tuning residues and assessed for sequence conservation.

## Supplementary Material

msaf158_Supplementary_Data

## Data Availability

The datasets generated and/or analyzed during the current study are available in the following Figshare repository: https://doi.org/10.48420/28856486.
